# Muscular and metabolic responses to different Nordic walking techniques, when style matters

**DOI:** 10.1371/journal.pone.0195438

**Published:** 2018-04-05

**Authors:** Barbara Pellegrini, Gennaro Boccia, Chiara Zoppirolli, Raffaela Rosa, Federico Stella, Lorenzo Bortolan, Alberto Rainoldi, Federico Schena

**Affiliations:** 1 CeRiSM Research Centre “Sport, Mountain, and Health”, Rovereto, (TN), Italy; 2 Department of Neuroscience, Biomedicine and Movement, University of Verona, Verona, (VR), Italy; 3 NeuroMuscularFunction | Research Group, School of Exercise and Sport Sciences, Department of Medical Sciences, University of Torino, Torino, (TO), Italy; University of L'Aquila, ITALY

## Abstract

Due to poling action and upper body engagement, Nordic walking (NW) has additional health benefits with respect to conventional walking. The aim of this study was to evaluate the differences in muscle activation and metabolic responses between NW, performed with the technique suggested by NW instructors, and with some modifications in the way to move upper limb and poles. Ten NW instructors volunteered to walk on a treadmill at 5.5 km•h^-1^ in five conditions: walking (W), Nordic walking (NW), NW with a weak poling action (NW_weak_), with straight-upper limbs moving the shoulders (NW_shoulder_) and with elbow flexion-extension pattern and shoulder freezed (NW_elbow_). Poling forces, body segments and poles movement, upper and lower body muscle activation, as well as metabolic parameters were measured.All modified NW techniques elicited lower muscular activation and metabolic responses with respect to the suggested NW technique (P < 0.05). All NW techniques elicited higher muscular activation and metabolic responses than W. All parameters observed with the NWweak were lower than NW. A decreased activation of shoulder extensor muscles and increased activation of anterior deltoid muscle were the main features of NW_shoulder._ Lower triceps brachii muscle activation and reduced propulsive poling action with respect to NW were seen for NW_elbow_, resulting also in shorter steps.Nordic walking instructors, sport technicians and practitioners should be aware that any deviation from the technique usually suggested might lead to lower benefits. However it is worth to note that any walking technique with poles elicits higher metabolic responses and muscular activation than walking.

## Introduction

Nordic walking (NW) is a form of physical exercise obtained by including the use of walking poles to conventional walking (W). The use of the poles actively engages the upper body to propel the body forward during walking, resulting in a higher activation of upper body musculature [1–3] and a greater energy expenditure with respect to walking, at a given speed [2, 4–7]. Due to these features, NW has become in the last 20 years a popular form of physical exercise. Several scientific contributions demonstrated health related benefits of NW on healthy adults [8, 9] elderly [10], overweight subjects [6, 11] and people affected by a wide range of pathologies [12]. Physical activity programs based on Nordic Walking were found to improved exercise capacity, functional status and quality of life [8, 12], by improving cardiorespiratory outcomes and lipidic profile, reducing body weight and chronic pain. On the other hand the effects of NW on muscle strength are still less clear [8, 12].

The Nordic Walking diagonal technique suggested by INWA (International Nordic Walking Association) [13] emerged from a training modality that is typical of cross country skiing. It is adopted and taught all over the world. This technique requires specific criteria: moving the arms respecting the range of movement of natural walking, maintaining a backward pole position during the loading phase, using the poles actively and dynamically and controlling the poles by hand gripping with a grasp/release pattern. The execution of the technique NW considered as correct is not obvious and automatic and, as noticed by both NW instructors and researchers [6, 14], the motion of the arms and the use of the poles often vary from those recommended. This deviation from the suggested technique may be caused by poor motor skill or insufficient training, self-learning or impairments at musculoskeletal or neuromuscular level [15]. Independently from the specific reason of deviation from the suggested NW technique, it is possible to hypothesize that differences in the gesture would affect the force applied through the poles, and both factors could lead to completely different patterns of upper body muscle activation and energy expenditure response to exercise.

An investigation analyzing specific poling techniques showed that different poling strategies influence lower limb gait mechanics [16]. In particular, when the poles were angled backward, greater push off impulse and ankle moments are required. Nordic walking style was found to influence pelvic movement and muscle activities of adults with hip osteoarthritis [17]. The pelvic rotation angle was significantly smaller and hip abductor muscle activity was significantly decreased in Japanese NW style, performed with poles held vertically, than in the more conventional NW style, here called “European” NW style where poles are kept diagonally inclined, with the top of the pole advanced with respect to the tip [17].

Most studies reported an increase in oxygen consumption between 18% and 25% in NW with respect to walking at the same speed [18, 19],[2, 6]. It is known that the increase of energy expenditure in NW changes according to many factors, such as terrain inclination [2, 5]and pole length [5]. Even though it has not yet investigated if differences in NW technique influence physiological responses to exercise, some papers suggest this hypothesis with their results [5, 6]. In one study it was seen that improved proficiency in NW technique increased the difference in energy expenditure between walking and NW [6]. Hansen and colleagues [5] found that when participants are instructed to perform a particularly vigorous NW technique, characterised by long steps and pole thrust, oxygen uptake exceed that measured during walking by 67%.

The aim of this investigation was to compare the biomechanical, muscular and metabolic responses elicited by NW with the suggested technique or with different techniques. We hypothesize that adopting altered techniques leads to diminished benefits arising from NW in terms of muscular activation and energy expenditure.

## Methods

### Participants

The study population was 10 (5 males, 5 females) NW instructors (mean age 37.7±12.0 years, height 1.70±0.08 m, body weight 61.5±7.3 kg) licensed by the ANWI (Associazione Nordic Walking Italia) with at least 2 years of experience in NW (mean 2.9±1.0 years) and having a lean body (mean body mass index 21.5±1.8 kg/cm^2^). The general health status was normal; none had any health condition that could affect exercise capacity. Participants were instructed to refrain from performing strenuous physical activity in the 24 h before the experimental session. All the participants provided their written informed consent before participating in the experiment. The study was approved by the Ethical Committee of Verona University and performed in accordance with the Helsinki Declaration.

### Overall design

Before starting the protocol, the subjects were prepared for EMG, kinematic and metabolic measurements. Participants were then requested to perform five minutes of exercise for each of the five NW techniques, on a motorized flat treadmill. The speed was set at 5.5 km/h as it was the most common in previous investigations, when subjects were free to select their speed of locomotion (healthy young and adults, [19] [20] [21] rehabilitation [22] elderly: [23]). Even though self selected NW speed is higher than self selected speed for conventional walking, [20] [21] [22], we decided to use the same speed for every condition, to avoid introducing a confounding factor in the study. The experimental conditions included conventional walking (W), Nordic Walking (NW), and three other locomotion with poles, in which the movement of arms and poles or the extent of poling force have been modified as described below. For NW condition, all the subjects were asked to use the diagonal-pole technique recommended by INWA [13]. According to the aforementioned technique, the correct use of the poles involves a backward pole position during the loading phase, active and dynamic use of the poles, and control of the poles with the grip and strap. The subjects were therefore requested also: to use the pole in a similar pattern of as the suggested technique, by however reducing the propulsive action exerted along the pole (NW_weak_); Nordic Walking with arm extended forward, trying not to flex the elbow and using therefore mainly shoulder joint (NW_shoulder_); to reduce the involvement of the shoulder, adopting therefore a technique in which arm motion, both during active poling an pole recovery phase is due mainly by flexion-extension of elbow joint (NW_elbow_). These three locomotion modes were shown by a national coach instructor to the subjects, who were allowed to practise until their technique was judged to be in line with the required one. To facilitate the subjects in performing the techniques required, they were allowed to see themselves during walking both on frontal and on sagittal plane. A mirror was hanged on the wall 4 m in front of the subjects and the image taken from a videocamera (Handicam, HDR-HC1E, Sony) placed laterally to the treadmill, was projected on a screen hanged just right to the mirror. The order of the trials was randomly selected for each participant.

Physiological measurements were taken throughout the five-minute duration of the trials, to verify the reaching of steady state in oxygen consumption and heart rate profile. However, only the last minute of exercise was used to evaluate the physiological demand of each type of exercise. For each trial, EMG and biomechanical data were acquired synchronously for 30 s during the last minute of exercise, to acquire the data of almost 10 consecutive stationary cycles. Subjects were allowed to rest and drink ad libitum between the trials and in any case the rest time lasted until their VO_2_ felt below a value of 10 ml/O_2_ per kg of body weight. Finally, participants were requested to perform two maximum voluntary isometric contractions (MVCs) for each muscle, which execution mode is further described in detail.

### Instruments and materials

#### Treadmill and poles

The experimental conditions were standardized in a biomechanically-equipped laboratory, by walking on a 2.5 x 3.5 m treadmill (Rodby Innovation AB, Vänge, Sweden). Comparability of actual speed and slope of the treadmill with nominal value is checked periodically. For the trials that required the use of the poles, the subjects used NW poles (Exel, Nordic Walker, Espoo, Finland) equipped with special carbide tips to ensure appropriate grip with the treadmill belt surface. As recommended by the INWA, correct pole length was determined by multiplying the subject’s height by 0.68 rounded down to the nearest 5 cm within a tolerance of 2.5 cm.

#### Metabolic measurements

A breath-by-breath metabolic cart (CPET, Cosmed, Rome, Italy) was used to measure the respiratory gas exchange values. A mask (70 ml dead space) was worn by the subjects to direct the respiratory gasses to the analyzers, through the sampling tube. A low-resistance bidirectional turbine, included in the mask, allowed the measurement of the inspiratory and expiratory volumes. Before each test, both turbine and gas analyzers were calibrated, as recommended by the manufacturers, the turbine through a 3-L volume syringe (Cosmed, Rome, Italy), the gas analyzers with ambient air (20.93% for oxygen and 0.03% for carbon dioxide) and a known concentration of gasses (16.00 ± 0.04% for oxygen and 5.00 ± 0.01% for carbon dioxide) (Air Liquide Italia S.p.A., Milano, Italia).

#### Kinematic measurements

A three dimensional optoelectronic motion capture system (Qualisys Pro-Reflex MCU 240, Qualisys AB, Gothenburg, Sweden), composed by 6 cameras put all around the treadmill, was used to acquire the coordinates of the markers with a sample frequency of 100 Hz. Before each test, the system was calibrated following the manufacturer’s guidelines. Calibration and data acquisition were performed using the specific software (Qualisys Track Manager) provided by the company. Fourteen reflective passive hemispherical markers were fixed on the skin of the subjects with double-sided tape. The markers were precisely positioned over the glenohumeral joints, humeral lateral condyles, ulnar styloids, greater trochanters, femoral lateral condyles, lateral malleolus and over the shoes, in proximity of the metatarsal head II. Furthermore, reflective tape was present in two sites of each pole to monitor their displacement.

#### Poling force measurements

A 15 g single-axial force transducer (custom built by Delta-tech, Sogliano al Rubicone, Italy) was inserted beneath the handgrip of each pole [24] to measure the force exerted through the poles during the trials that required the use of the pole. Before each test, the transducers were dynamically calibrated using a reference cell (546 QD; DS Europe srl, Milano, Italy) by simulating five poling actions against an external reference cell, to calculate the mean error of left and right force transducer measurements with respect to the reference. During the experimental trials, force signal was sampled at 200 Hz, by means of a data acquisition board (NI DAQ-Pad-6016, National Instruments, Austin, TX, USA). The cables coming from the transducers were kept adherent to the skiers’ arms through elastic nets, not to interfere with the poling movement.

#### Electromyographic measurements

EMG signals were recorded at a sample frequency of 2048 Hz using multichannel amplifier (EMG-USB2, OT Bioelettronica, Turin, Italy) with a recording bandwidth 10–500 Hz. Bipolar Ag/AgCl surface EMG electrodes (Spes Medica, Battipaglia, Italy), with 2-cm inter-electrode distance, were placed over 15 muscles of the right side of the body including: *Tibialis Anterior* (TA), *Soleus* (SO), *Gastrocnemius Medialis* (GM), *Vastus Lateralis* (VL), *Rectus Femoris* (RF), *Biceps Femoris* (BF), *Semitendinosus* (ST), *Gluteus Medius* (Glu), *Upper Trapezious* (UT), *Erector Spinae* (ES), *Latissimus Dorsi* (LD), *Anterior Deltoid* (AD), *Posterior Deltoid* (PD), *Biceps Brachii* (BB) and *Triceps Brachii* (TB). Before the placement of the electrodes, the skin was slightly abraded with abrasive paste and cleaned with water in accordance with SENIAM recommendations [25]. The optimal position and orientation of the electrodes were sought for each muscle following guidelines previously described [26]. A preliminary test was performed to check for cross talk and cable-induced noise and, when needed, electrodes and cables were repositioned and secured on the body with an extensible dressing (Fixomull^®^, Beiersdorf, Hamburg, Germany) to avoid movement artefacts. MVCs were performed against immovable resistance provided by an operator or by fixed tools. Joint angles or muscles length were selected to be as close as possible to those of the task investigated [27]. For TA, MVCs were obtained by dorsiflexion with subject seated, knee at 45°, heel on the floor. For GM and SO, MVC was obtained by ankle plantar flexion from ankle in neutral position, with subject standing, knee and hip straight, weight supported on tested side. For VL and RF, MVCs were obtained by knee extension, with the subject seated, knee at 45°. For BF and ST, MVCs were obtained by knee flexion, with subject in prone position, knee flexed at 20°. GLU MVC was obtained by leg abduction with straight hip and knee, with subject lying on the contralateral side. For UT, MVCs was obtained by shoulder elevation from neutral position, with subject seated, arm alongside the body. For ES, MVC was obtained by trunk extension in prone position. For LD, MVC was obtained by shoulder depression and extension with arm alongside the body, elbow slightly flexed, in seated position. For AD and PD, MVCs were obtained respectively by shoulder flexion and extension, with shoulder at 0° abduction, 0° flexion, elbow flexed at 45 °. For BB and TB, MVCs were obtained respectively by elbow flexion and extension, with shoulder at 0° abduction, 0° flexion, elbow flexed at 45 °.

### Data analysis

#### Physiological data processing

The average value of oxygen consumption expressed as ml/O_2_ per kg of body weight (V’O_2_), ventilation (VE) and heart rate (HR) were calculated over the last 60 s of each condition.

#### Biomechanical data processing

Gait cycles were identified for all conditions as the interval between two consecutive heel strikes of the right foot. Cycle time (CT) was therefore calculated as the time between two consecutive heel strikes of the right foot. Time of contact of the foot (FCT) was calculated as the interval between the heel strike to the time of metatarsal head take-off. Heel strike and metatarsal head take-off were identified by the maximum of forward displacement of the malleolus and by the vertical speed of metatarsal head, respectively [28]. The ratio between the time spent in foot contact with respect to the total time has been expressed by means of the duty cycle parameter for foot (DC_foot_ = FCT/CT *100).

Flexion extension movements have been calculated for elbow, shoulder, hip, knee and ankle joints on sagittal plane for both body sides. Maximum, minimum, and angular range of movement (ROM) values for each joint has been calculated. Where poles were present, pole inclination was calculated as the angle between the direction of the pole in sagittal plane and the horizontal plane. Maximum, minimum, range and average pole inclination was then calculated for each side for a minimum of 10 consecutive cycles and then averaged together. Pole contact and pole take-off were identified from the force data, as the first point above and the first point below a force threshold of 5 N, respectively. The duration of the poling action, called poling time (PT), has been calculated as the time between pole ground contact and pole take-off. The ratio between the time spent in the poling phase with respect to the total time has been expressed by means of the duty cycle parameter (DC_pole_ = (PT/CT)*100).

The total force applied to the pole was obtained for each cycle. The tangential force component representing the propulsive part of the poling force was calculated by multiplying the total force by the cosine of the pole inclination. For each cycle, the integral poling force over the entire cycle (PFtot) and the integral of propulsive part of the poling force over the entire cycle (PFprop) were calculated. The ratio between the propulsive component and the total force has been defined force effectiveness [29] E% = (PFprop /PFtot)*100 and was also calculated.

Each parameter described above has been calculated for each side and for 10 consecutive cycles and then averaged together.

#### Electromyographic data processing

Before EMG processing, the EMG signals were carefully inspected, checking for noise and movement artefacts. For each trial, 10 consecutive, non-corrupted stride cycles were selected for analysis. Raw EMG signals were band-pass filtered (bi-directional, 4th-order, zero lag Butterworth, band-width 20–400 Hz) to attenuate motion artefacts. All signals were then rectified and low-pass filtered at 9 Hz (bi-directional, 4th-order, zero lag Butterworth), resulting in the EMG envelopes [30]. EMG amplitude was normalized with respect to the EMG amplitude detected during the MVC manoeuvres. The EMG signals recorded during MVCs were filtered as described above, rectified, and averaged using 500 ms epochs of signal. The epoch with the highest value was then considered as the reference for the amplitude normalization of the EMG envelopes.

For visualization purposes, the EMG signals for each gait cycle, was time-normalized to 100 points, and averaged EMG profile over 10 cycles and all subjects was calculated for each condition. The average value of normalized EMG profiles (i.e. average EMG signal amplitude) for each subjects and condition was then used a measure of muscle activation throughout the gait cycle.

### Statistical analysis

Kolmogorov-Smirnov normality test was used to assess distributions normality. If the data were not normally distributed were log-transformed before entering the statistical analysis. Repeated measure Analysis of Variance (ANOVA) was used to compare the kinematic, metabolic and electromyographic values across conditions. When data sphericity was violated, Greenhouse-Geisser correction was applied. When ANOVA resulted statistically significant, Dunnett’s post hoc tests were then used to compare W values with respect to the other conditions. A second Dunnett’s post hoc test was conducted to compare NW values with respect to other conditions. Threshold for statistical significance was set to p<0.05. Statistical analyses were performed with SPSS statistics (version 20.0, IBM Corporation, Somers, NY). Data are all expressed as mean±SD. The magnitude of the effect of the condition was calculated as partial eta squared: >0.01 small, >0.06 medium, >0.14 large [31].

## Results

### Kinematics

The pattern of motion in the different technical conditions performed by a representative subject is visible in videos included as supplementary files (S1 Video; S2 Video, S3 Video, S4 Video, S5 Video). Cycle time and FCT changed significantly across the different locomotions, and the magnitude of effect was large, (Table 1). Cycle time increased, with respect to W, in NW, NW_weak_ and NW_shoulder_ by 8.4%, 6.0% and 4.9% respectively; no differences were found between W and NW_elbow_. DC_foot_ showed no condition effects.

**Table 1:** Mean ±SD of kinematic data.

	W	NW_weak_	NW_shoulder_	NW_elbow._	NW	F	p	η2
CT [s]	1.03±0.04***	1.08±0.06###	1.09±0.06###	1.06±0.04***	1.12±0.05###	15.0	**P<0.0001**	0.625
FCT [s]	0.61±0.03***	0.65±0.05*,###	0.65±0.05*,###	0.63±0.04***	0.68±0.05###	18.3	**P<0.0001**	0.670
DF_foot_ [%]	59.71±1.41	59.67±1.33	59.49±1.40	59.33±1.27	59.67±1.64	0.96	0.4394	0.097
PT [s]	-	0.53±0.10**	0.57±0.06	0.52±0.04***	0.60±0.06	8.92	**0.0003**	0.498
DF_pole_ [%]	-	48.6±6.5**	51.8±2.8	50.2±3.5	52.3±3.1	4.35	**0.013**	0.326
HIP_max_ [°]	195±3	195±4	195±4	195±5	195±3	0.44	0.781	0.046
HIP_min_ [°]	158±5***	155±5 #	156±6	155±6 #	154±6 ###	4.76	**0.004**	0.346
HIP_ROM_ [°]	36±3***	40±4 ##	39±4 ##	40±3 ##	41±4 ###	7.55	**0.0002**	0.456
KNEE_max_ [°]	172±4	172±4	172±4	172±4	172±4	0.69	0.602	0.071
KNEE_min_ [°]	110±3*	111±4	112±5	110±3*	113±5 #	3.79	**0.011**	0.296
KNEE_ROM_ [°]	62±3	60±3	60±3	62±4	60±4	2.58	0.054	0.223
ANKLE_max_ [°]	126±6	126±6	125±7	126±7	126±6	0.46	0.764	0.124
ANKLE_min_ [°]	91±3	90±3	91±3	90±2	90±3	1.27	0.301	0.049
ANKLE_ROM_ [°]	35±5	36±6	35±6	36±6	36±6	1.55	0.210	0.147
SHOULDER_max_ [°]	21±13	20±11	44±8***;#	7±7**;#	21±12	29.27	**P<0.0001**	0.765
SHOULDER_min_ [°]	-29±8	-19±9**;#	-1±8***;#	-15±5***;#	-30±11	30.28	**P<0.0001**	0.771
SHOULDER_ROM_ [°]	49±20	39±13 #	45±8	22±3***,#	51±11	12.43	**P<0.0001**	0.580
ELBOW_max_ [°]	164±10	153±12 *;#	158±8	143±11***; #	162±6	17.09	**P<0.0001**	0.655
ELBOW_min_ [°]	126±11*	112±21 #	136±9***	86±16 ***;#	115±16 #	39.61	**P<0.0001**	0.815
ELBOW_ROM_ [°]	38±10*	42±13	22±8***,###	57±11*,###	47±13#	27.95	**P<0.0001**	0.756
POLE_max_ [°]	-	66±8	70±4*	75±6***	65±6	11.76	**0.000**	0.566
POLE_min_ [°]	-	36±3	37±3**	40±3***	34±4	9.98	**0.001**	0.526
POLE_ROM_ [°]	-	30±8	33±6	35±6*	31±7	5.90	**0.003**	0.396

Statistically significant difference from conventional walking (W) are reported as ^#^ p<0.05, ^##^ p<0.01, ^###^ p<0.001.

Statistically significant difference from Nordic walking (NW) are reported as * p<0.05, ** p<0.01, *** p<0.001

Lower limb kinematics showed a significant and large effect of locomotion for minimum angle of hip, hip ROM and minimum angle of knee (Table 1). Post hoc test revealed significant differences between W and NW for all the above-mentioned parameters, and a difference between W and all NW conditions for hip ROM.

All parameters referring to upper-body and pole kinematics shown a significant and large effect of the locomotion (Table 1). Post hoc test revealed that significant differences between W and NW were found only for minimum of elbow angle and elbow ROM, showing a decrease of 8.9% and an increase of 23.8% respectively. In NW_shoulder_ maximal shoulder angle increased by 114% and minimal shoulder angle decreased by 95% with unchanged shoulder ROM with respect to NW. Moreover, an 18% increase in elbow extension and 52.4% decrease in elbow ROM was found for NW_shoulder_ with respect to NW. Post hoc test showed for NW_weak_ a 38.8% and 24.2% reduction in shoulder minimal and ROM angle respectively and a 5.4% decrease in elbow maximal extension with respect to NW. NW_elbow_ was seen to change all shoulder and elbow parameters, with significant decrease of minimal and maximal values for both joints with respect to NW, and with a 57.5% decrease of shoulder ROM and a 20.3% increase in elbow ROM.

### Poling action

Pole inclination was significantly affected by NW technique (Table 1). Post hoc showed an increase in maximal and minimal inclination of pole for NW_shoulder_ and NW_elbow_ and a 15.8% increase in pole ROM for NW_elbow_ with respect to NW.

**Fig 1:**
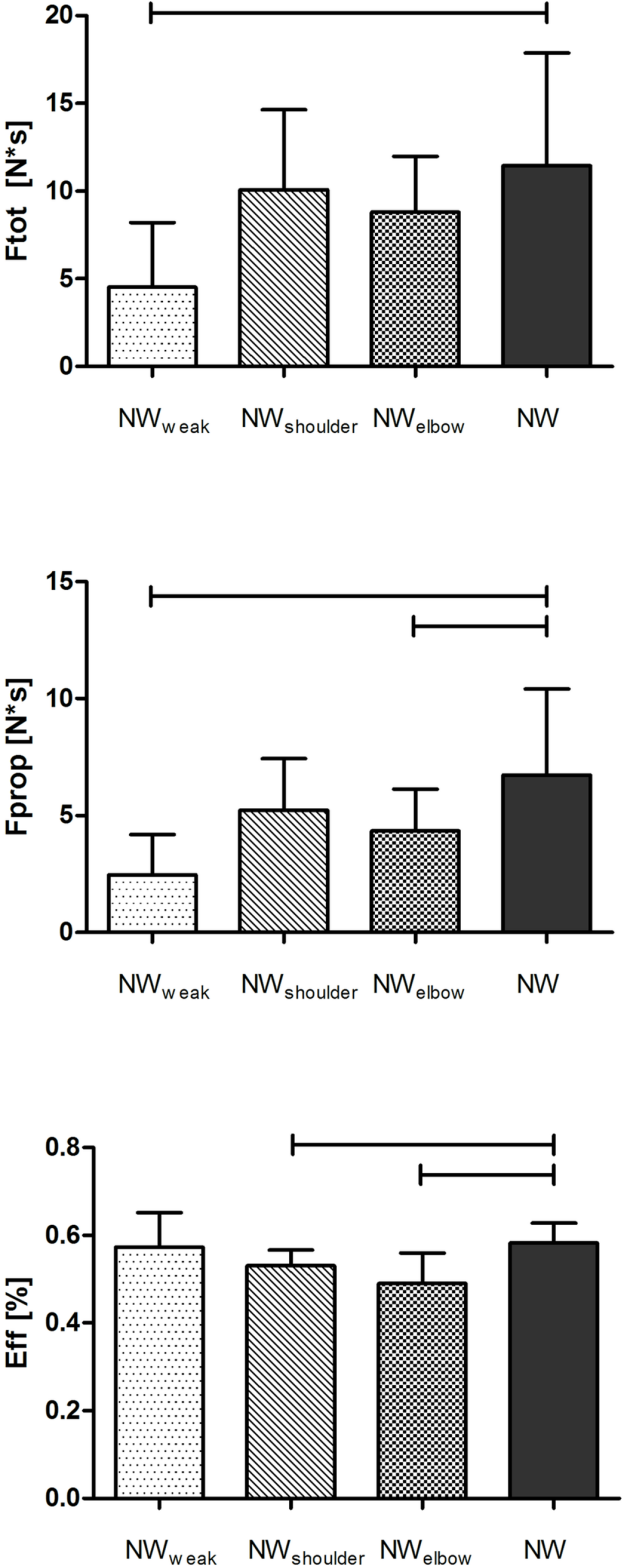
Mean values for parameters of total poling force (Ftot, upper panel), propulsive component of poling force (Fprop, central panel) and force effectiveness (EFF%, bottom panel). The error bars indicate standard deviation. Horizontal bars indicate significant differences between NW and other conditions (p < 0.001).

A significant and large effect of NW technique was seen in poling time and poling duty factor (Table 1). PT decreases by 12% and 14.2% for NW_weak_ and NW_elbow_ with respect to NW; DC_pole_ decreased by 7.1% for NW_weak_ with respect to NW. Poling force has a significant and large condition effect (F(3,27) = 27.95, p<0.0001, η^2^ = 0.569) and was 60.5% lower for NW_weak_ with respect to NW (Fig 1A) Propulsive component of force was significantly and largely affected by technique, (F(3,27) = 13.12, p<0.0001, η^2^ = 0.593) and pot hoc test revealed a significant decrease in F_prop_ for NW_weak_ and NW_elbow._ by 63.4% and 35.5% respectively (Fig 1B). A significant and large effect of NW technique was found for Eff (F(3,27) = 13.19, p<0.0001, η^2^ = 0.594) and pot hoc showed this parameter is 10.7% and 17.3% lower for NW_shoulder_ and NW_elbow_ respectively with respect to NW (Fig 1C).

### Muscle activity

ANOVA analysis revealed a significant technique effect on the EMG averaged rectified values of the following three muscles (GM, BF and GM) out of eight lower body muscles, and in every upper body muscle except for ES (Table 2).

**Table 2:** Mean (±SD) of EMG envelopes normalized by the maximum voluntary contraction (%).

	W	NW_weak_	NW_shoulder_	NW_elbow_	NW	F	p	η^2^
Tibialis Anterior	14.4±1.4	12.8±2.2	13.3±2.0	13.4±2.3	13.7±2.1	2.14	0.095	0.214
Soleus	8.2±1.6	8.7±1.9	6.9±1.1	7.3±1.0	8.5±2.5	2.43	0.065	0.213
Gastrocnemius Medialis	8.3±0.8	7.5±1.9	6.0±1.2^#^	6.8±2.3	7.7±1.5	4.58	**0.004**	0.337
Vastus Lateralis	5.8±1.4	6.2±1.6	6.5±1.1	6.2±1.5	8.0±3.9	2.33	0.074	0.206
Rectus Femoris	3.7±1.1	3.3±1.2	3.8±1.8	2.9±0.9	2.9±1.1	1.64	0.185	0.154
Biceps Femoris	6.1±2.3*	4.5±2.2	5.8±1.9	4.4±1.3	4.3±2.0^#^	3.13	**0.026**	0.259
Semitendinosus	8.3±3.7	11.1±7.2	8.1±3.4	7.8±3.5	6.6±2.1	2.72	**0.044**	0.233
Gluteus Medius	10.2±2.8	9.9±4.4	7.8±2.2	7.4±2.1^#^	8.4±3.3	2.48	**0.047**	0.216
Upper Trapezius	3.8±1.2	7.0±4.2^#^	6.8±3.3^#^	4.3±1.8	5.5±3.6	3.95	**0.009**	0.305
Erector Spinae	6.9±2.2	6.3±2.5	7.3±2.1	6.5±1.8	6.6±1.6	0.769	0.552	0.079
Latissimus Dorsi	3.0±1.0***	3.6±1.1***	6.7±2.3^#^,**	8.5±4.3^###^	11.1±6.3^###^	13.21	**<0.0001**	0.595
Anterior Deltoid	1.2±0.4*	2.1±0.3	5.8±2.0^###^,***	2.2±0.6	2.4±1.0^#^	35.65	**<0.0001**	0.798
Posterior Deltoid	3.3±1.4***	4.6±1.5***	8.2±3.9^###^,***	5.9±2.6***	14.1±6.0^###^	25.96	**<0.0001**	0.743
Biceps Brachii	1.3±0.4***	3.4±1.8^###^	3.3±1.4^##^	4.3±2.1^###^	3.5±1.6^###^	9.27	**<0.0001**	0.507
Triceps Brachii	2.3±0.8***	5.5±3.2^#^,***	6.7±2.6^##^,***	8.0±3.2^###^,***	13.8±6.4^###^	26.02	**<0.0001**	0.743

Statistically significant difference from conventional walking (W) are reported as ^#^ p<0.05, ^##^ p<0.01, ^###^ p<0.001.

Statistically significant difference from Nordic walking (NW) are reported as * p<0.05, ** p<0.01, *** p<0.001.

Averaged activation of lower body muscles was in general lower than 15% of the MVC values. Post hoc test showed significant differences between W and some of the NW conditions. Specifically, activation of GM resulted 38% higher in W than NW_shoulder_; activation of BF resulted 29% higher in W than in NW and activation of Glu resulted 27% higher in W than in NW_elbow._

Upper body muscles showed EMG values (normalized to MVC) generally lower than leg muscles. EMG higher than 10% with respect MVC was seen in NW in the following three upper limb muscles: LD (NW 11.1 ± 6.3%, all other conditions ≤ 8.5%); PD (NW 14.1 ± 6.0%, all other conditions ≤ 8.2); and TB (NW 13.8 ± 6.4%, all other conditions ≤ 8.0%). These muscles were indeed involved in the poling phase. The involvement of other muscles reached value lower than 9% of MVC in every condition. ANOVA showed a significant and large effect of locomotion for upper body muscles except for ES. EMG of UT was 84% and 78% higher for NW_weak_ and NW_shoulder_ respectively than for W. EMG of LD was higher than for W in NW_shoulder_, NW_elbow_ and NW. In NW, EMG from LD was 208% and 65% higher than for NW_weak_ and NW_shoulder_ respectively_._ EMG of AD was maxima in NW_shoulder,_ being 141% and 383% higher than for NW and W respectively. EMG of AD was 100% higher in NW than in W. EMG of PD and TB was the highest for NW, and was significantly higher for NW with respect all other conditions. TB activity was in all the pole locomotion higher than for W, PD activity was higher than for W for NW and NW_shoulder._ EMG for BB was higher than for W in all pole locomotion. No differences between NW and the other pole walking techniques were seen.

**Fig 2:**
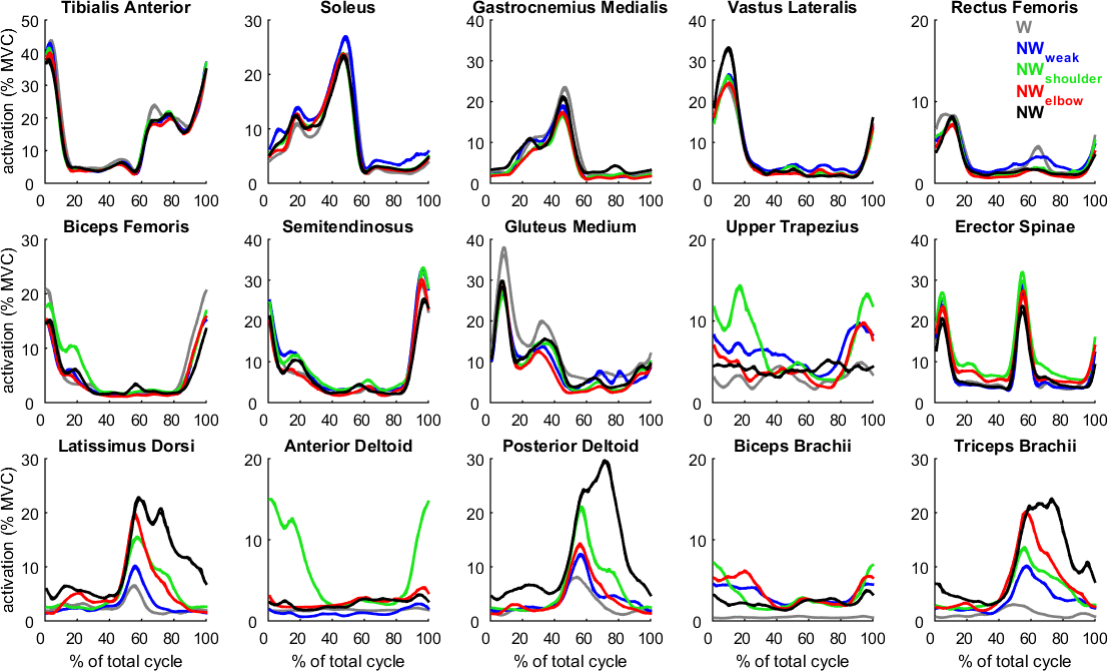
Group-averaged envelopes of electromyographic signals, normalized with respect to the maximum voluntary contraction (MVC), are reported for 15 recorded muscles for conventional walking (grey), NW_weak_ (blue), NW_shoulder_ (green), NW_elbow_ (red), and NW (black). Each represented pattern consists in the average of 10 cycles of the eleven subjects.

The average EMG envelopes are illustrated in Fig 2. All lower limb and trunk muscles showed similar shape of envelopes across conditions. Conversely, upper limb muscles (bottom row of the Fig 2) were mostly affected by the conditions. LD, PD and TB not only showed higher activation for NW if compared to the other conditions, but also showed a delayed and longer activation time. AD showed a particular shape, with high activation detected mainly during pole recovery action and a short activation during the last part of poling action. The average EMG envelopes suggest that when a decreased activation was found, this is likely due to a decrease of both duration and intensity of activation.

### Metabolic responses

**Fig 3:**
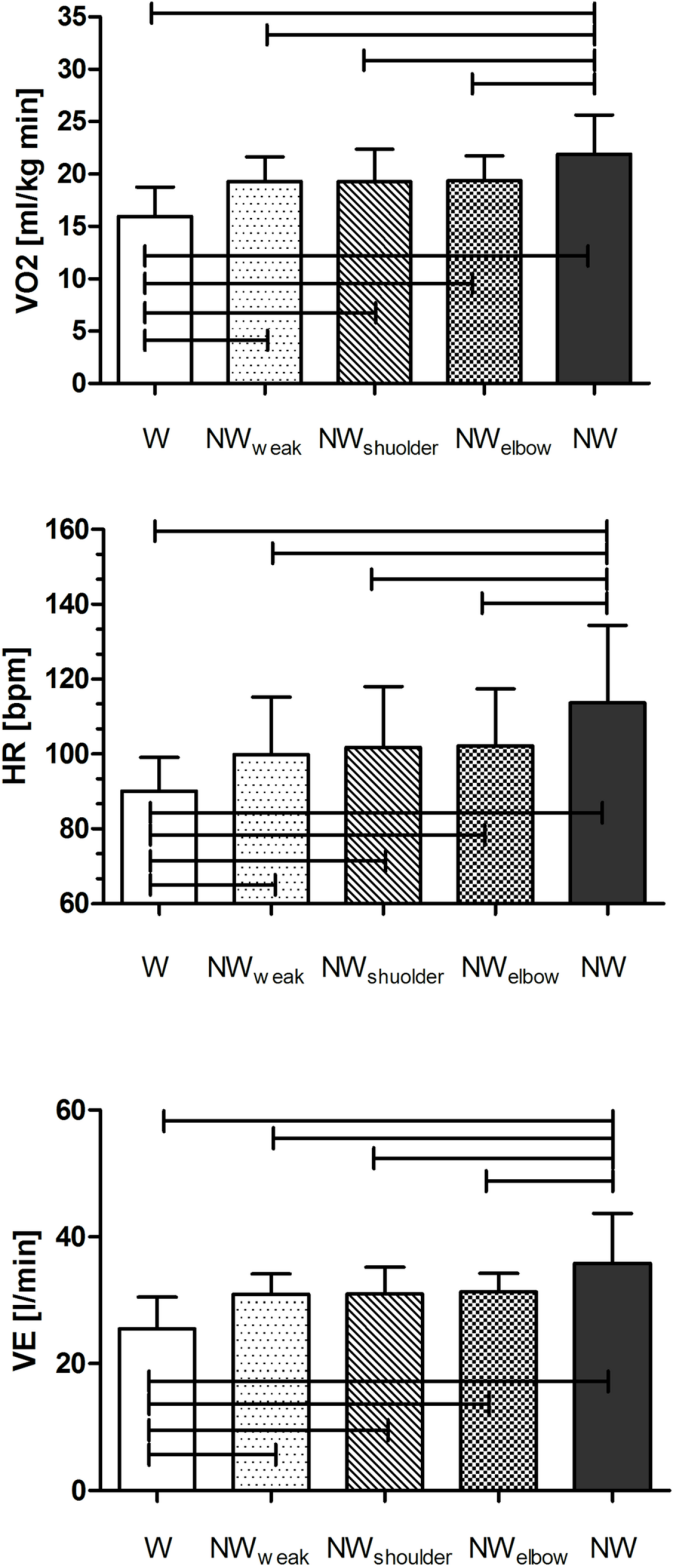
Mean values for VO_2_ (upper panel), HR (central panel) and ventilation (VE, bottom panel) for each conditions. The error bars indicate standard deviation. Horizontal bars indicate significant differences between W and all other conditions and between NW and all other conditions (p < 0.001).

A significant effect of locomotion was found for all the metabolic parameters measured (F(4,36) = 22.17, p<0.0001, η^2^ = 0.711 for VO_2,_ F(4,36) = 12.68, p<0.0001, η^2^ = 0.585 for HR and F(4,36) = 13.70, p<0.0001, η^2^ = 0.604 for VE (Fig 3).

Post hoc test showed a significant increment of VO_2_ for all the NW techniques with respect to W, with the highest difference found for NW with respect to W, +37%. With respect to NW, VO_2_ for NW_weak_, NW_shoulder_ and NW_elbow_ decreased by 11.9%, 12.0% and 11.7% respectively.

Post hoc test showed that HR was significantly higher in NW than in W (+23 bpm). Significant differences were seen also between W and all others walking with pole conditions, with HR being on average 11.6, 12.1 and 23.5 bpm higher in NW_weak_, NW_shoulder_ and NW_elbow_ respectively. With respect to NW, HR was on average 13.8, 11.9 and 11.43 pbm lower in NW_weak_, NW_shoulder_ and NW_elbow_ respectively.

Post hoc test for VE showed a significant increment for all the walking with pole conditions with respect to W. With respect to NW, VE was 13.5%, 13.3% and 12.5% lower for NW_weak_, NW_shoulder_ and NW_elbow_ respectively.

## Discussion

The data of our study confirm the results of previous investigations showing biomechanical changes, an overall higher activation of upper body muscles and energy expenditure when using walking poles with respect to conventional walking [1, 2, 7].

The most important finding is that, when walking with the pole is performed by adopting different techniques from that suggested as the optimal, it brings to a reduction of the muscular and metabolic responses with respect to NW. In any case, walking with poles has been found, regardless to the technique used in this investigation, to elicit higher physiological and muscular responses with respect to conventional walking.

In this investigation, the Nordic Walking style suggested by INWA was taken as the reference technique to perform Nordic Walking. This association is indeed known over the world, having affiliations in 20 nations, and the INWA technique have been utilized in other scientific investigations [2, 6, 7, 10, 32, 33].

When NW was performed with the suggested style, we found significant increments of upper body muscle activation and an increase in oxygen consumption, confirming the results of previous investigations [2, 7]. From a kinematical point of view, when performed with the suggested technique, NW only slightly alters the biomechanical pattern of conventional walking, in line with the declared aim of INWA association. With respect to walking induced a 3- to 5-fold higher activation of the muscles involved in the poling action and a twofold higher activation of the muscle devoted to the pole recovery. The increments of energy expenditure for NW found in this investigation exceeded by 37% that for conventional Wand was therefore even greater than those in previous similar studies, where an 15–25% increase was found [2, 6, 19]. This discrepancy may be explained by the fact that instructors in our investigation were engaged in a particularly vigorous technique. In a similar scenario, Hansen and colleagues [5] measured an increase of energy expenditure of 67% with respect to conventional walking.

When NW was performed with a “weak” style, the upper body and pole kinematics remained substantially unaltered with respect to suggested NW, however, the activation of muscles involved in the poling action, *latissisum dorsii*, *posterior deltoid* and *triceps brachii*, substantially decreased with respect to regular NW This resulted in a 60% decrease in the force exerted through the pole. Despite this, holding the poles and actively swinging the upper limbs lead to an activation of *biceps brachii* and *upper trapezius involvement*, which was similar to those recorded during suggested NW. Therefore, the weak style NW, even if it does no lead to a great involvement of muscle activity, significantly enhanced intensity of exercise and still brings some benefits with respect to conventional walking. However, when weak style is compared to suggested NW technique, a 10% lower VO_2_ and 14 bpm lower heart rate response are found. Therefore, performing a weak style NW rather that the suggested technique could decrease the efficacy of training program. This can be relevant when fit people perform NW; indeed it has been demonstrated that Nordic walking on flat surface is not intense enough to reach high training zone in young and fit subjects [34]. When subjects were asked to use Nordic poles by keeping the arms extended the pattern of arm motion resemble those of other pole walking technique in which, a higher arm position, and a continuous hand grip is suggested [15]. When have found that muscular activity resulted completely different from those seen when correct NW is performed. All muscles involved in poling action, *triceps brachii*, *posterior deltoid* and *latissimus dorsii*, decreased their activity with respect to correct NW. *Anterior deltoid* muscle conversely increased its engagement more than twice with respect to regular NW. Exercising therefore with poles by keeping the arms straight and high lead thus an increased engagement of shoulder flexor muscles and a decreased use of shoulder and elbow extensors … An excessive activation of muscles controlling shoulder flexion, together with a low activation of antagonist back muscles may negatively increase shoulder protraction, one of the most common postural alteration in people of all ages [35]. On the contrary, a balanced muscle activation may be beneficial in chronic non-specific pain reduction [36]. It was found indeed that performing NW with suggested technique, may lead to a reduction of neck pain in patients [37].

The energy expenditure associated with Nordic Walking with arm extended straight forwards was 12% lower than those for regular NW, however still 21% higher than those for conventional walking. From a cardiovascular point of view therefore instructors and practitioners should be aware that this deviation from suggested technique could lead exercise intensity to fall below the training zone expected for NW.

When subjects were asked to use poles mainly by flex-extending the elbows and maintaining the shoulders locked, poles were kept more vertically and a substantial reduction in propulsive component of poling force was measured. With this style the step length was lower than that observed for regular NW. In NW the increase of step length could be ascribed either to the propulsive action exerted trough the pole and to the longer time required to complete a wide arm and pole swing. Both these features were lower for the technique discussed here and may have concurred in preventing step lengthening. Using poles in vertical direction and working only with elbow, could therefore limit the improvement in step length that could be obtained in Nordic walking fitness programs [38]. Moreover, with this style *gluteus medius* decreased by 27% its activation with respect to conventional walking. This is somehow in agreement with a study that demonstrated that when the walkers use the pole vertically, like a cane, this brings to a reduction in the activation of *gluteus medius*, *gluteus maximus* and *tensor of fascia latae* muscles, with respect to conventional walking [17]. Lower hip abductor muscle activity could be attributed to the ground reaction force elicited by the pole against gravity, which complements the activity of the hip abductors. This may be considered as protective for subjects suffering from hip joint disease, as ostheoartithes. This NW style lead however to a decreased engagement of *posterior deltoid* and *triceps brachii* muscles, with respect to regular NW., *Latissimus dorsii* activation remained similar to those of regular NW despite small shoulder range of movement. and could be attributed more to joint freezing than to shoulder extension action [39]. Physiological responses were 21% higher than for conventional walking, however 12% lower with respect to regular NW. This style may therefore be used when improving stability and hip protection is desired, having however in mind that lower muscular and metabolic responses are elicited.

This investigation has been conducted on NW instructors. This choice, that is common in similar investigation on NW, ensures that subjects perform a good NW technique and have the skill to precisely execute the requirements of the investigators. On the other hand, it brings limitations too. First, the low number of subjects involved decreases the ability of the experimental design in detecting small effect size. This means that possible small differences between the styles may be not detected in this investigation. Nevertheless, in everyday practice small changes may be not impactful and significant effects detected and discussed here are still valid. Due to the rather low number of subjects, we avoided to include psycho-physical parameters as rate of perceived exertion that, due to their low signal to noise ratio, would have suggested a higher sample size [40]. Secondly, the involvement of a population of healthy and fit subject decreases the external validity. Despite of this, we could not do otherwise, as we would like to compare different styles. We have of course to be aware that the generalization of the results of this study to a wider population should be made with caution.

With this work, we investigated some deviations from the suggested Nordic Walking technique. The conditions tested here are however not exhaustive with respect to all the possible conditions in real Nordic Walking practise. We did not test the responses to altered technical pattern combined with a weak poling action. We indeed created different conditions by modifying one feature at a time; we therefore kept the poling force unaltered with respect to regular NW for all the condition but for “weak action”. We can however speculate that combination of weak action and altered movement pattern would lead to an even lower response with respect to those seen in our investigation.

## Conclusion

When Nordic Walking is performed with the suggested technique, it allows reaching higher metabolic intensities and generally higher muscling activations. Greater acute response to correct Nordic Walking technique demonstrated here lead to hypothesise that by controlling and correcting individual technique through a period of training may lead to enhance the benefits coming from its practise. Based on the results of our study we recommend that healthy people should make an effort in performing Nordic Walking with the technique considered as the correct one. As previously suggested, the present findings furthermore support the importance of being supervised in skill learning in order to reach the optimal technique execution.

It is worth noting however that, even when walking with pole is performed adopting a technique different from that considered the correct one, this can still lead to metabolic responses and muscle activations that are greater than those of conventional walking. This would suggest that even in case the Nordic Walking style cannot resemble those recommended, benefit superior to those of conventional walking could still be reached.

## Supporting information

S1 Video Animation data illustrate three consecutive cycles of subject 4 for walking. The dotted lines represent the left side and the continuous lines represent the right side. Use “repeat” function on the movie player to see the stick walking continuously.
(MOV)

S2 Video Animation data illustrate three consecutive cycles of subject 4 for NW_weak_ performed with suggested technique. The dotted lines represent the left side and the continuous lines represent the right side. Poling force for left and right side represented by red and blue bar draw on the respective poles with length proportional to force value. Use “repeat” function on the movie player to see the stick walking continuously.
(MOV)

S3 Video Animation data illustrate three consecutive cycles of subject 4 for NW_shuolder_ performed with suggested technique. The dotted lines represent the left side and the continuous lines represent the right side. Poling force for left and right side represented by red and blue bar draw on the respective poles with length proportional to force value. Use “repeat” function on the movie player to see the stick walking continuously.
(MOV)

S4 Video Animation data illustrate three consecutive cycles of subject 4 for NW_elbow_ performed with suggested technique. The dotted lines represent the left side and the continuous lines represent the right side. Poling force for left and right side represented by red and blue bar draw on the respective poles with length proportional to force value. Use “repeat” function on the movie player to see the stick walking continuously.
(MOV)

S5 Video Animation data illustrate three consecutive cycles of subject 4 for NW performed with suggested technique. The dotted lines represent the left side and the continuous lines represent the right side. Poling force for left and right side represented by red and blue bar draw on the respective poles with length proportional to force value. Use “repeat” function on the movie player to see the stick walking continuously.
(MOV)

## References

[1] Schiffer T, Knicker A, Montanarella M, Struder HK. Mechanical and physiological effects of varying pole weights during Nordic walking compared to walking. Eur J Appl Physiol. 2011;111(6):1121–6. doi: 10.1007/s00421-010-1739-5 21113789 10.1007/s00421-010-1739-5

[2] Pellegrini B, Peyre-Tartaruga LA, Zoppirolli C, Bortolan L, Bacchi E, Figard-Fabre H, et al Exploring Muscle Activation during Nordic Walking: A Comparison between Conventional and Uphill Walking. PLoS One. 2015;10(9) 10.1371/journal.pone.0138906 PMC4587792 26418339

[3] Shim JM, Kwon HY, Kim HR, Kim BI, Jung JH. Comparison of the Effects of Walking with and without Nordic Pole on Upper Extremity and Lower Extremity Muscle Activation. J Phys Ther Sci. 2013;25(12):1553–6. doi: 10.1589/jpts.25.1553 24409018 10.1589/jpts.25.1553 PMC3885837

[4] Schiffer T, Knicker A, Hoffman U, Harwig B, Hollmann W, Struder HK. Physiological responses to nordic walking, walking and jogging. Eur J Appl Physiol. 2006;98(1):56–61. doi: 10.1007/s00421-006-0242-5 16799817 10.1007/s00421-006-0242-5

[5] Hansen EA, Smith G. Energy expenditure and comfort during Nordic walking with different pole lengths. J Strength Cond Res. 2009;23(4):1187–94. doi: 10.1519/JSC.0b013e31819f1e2b 19528847 10.1519/JSC.0b013e31819f1e2b

[6] Figard-Fabre H, Fabre N, Leonardi A, Schena F. Physiological and perceptual responses to Nordic walking in obese middle-aged women in comparison with the normal walk. Eur J Appl Physiol. 2010;108(6):1141–51. doi: 10.1007/s00421-009-1315-z 20091181 10.1007/s00421-009-1315-z

[7] Sugiyama K, Kawamura M, Tomita H, Katamoto S. Oxygen uptake, heart rate, perceived exertion, and integrated electromyogram of the lower and upper extremities during level and Nordic walking on a treadmill. J Physiol Anthropol. 2013;32(1) 10.1186/1880-6805-32-2 PMC3599214 23406834

[8] Fritschi JO, Brown WJ, Laukkanen R, van Uffelen JGZ. The effects of pole walking on health in adults: A systematic review. Scand J Med Sci Spor. 2012;22(5):e70–e8 10.1111/j.1600-0838.2012.01495.x 22734947

[9] Mathieson S, Lin CWC. Health benefits of Nordic walking; a systematic review. Brit J Sport Med. 2014;48(21):1577–8. 10.1136/bjsports-2013-093294 24505040

[10] Parkatti T, Perttunen J, Wacker P. Improvements in Functional Capacity From Nordic Walking: A Randomized Controlled Trial Among Older Adults. J Aging Phys Activ. 2012;20(1):93–105. 10.1123/japa.20.1.93 21949243

[11] Figard-Fabre H, Fabre N, Leonardi A, Schena F. Efficacy of Nordic walking in obesity management. Int J Sports Med. 2011;32(6):407–14. doi: 10.1055/s-0030-1268461 21472629 10.1055/s-0030-1268461

[12] Tschentscher M, Niederseer D, Niebauer J. Health benefits of Nordic walking: a systematic review. Am J Prev Med. 2013;44(1):76–84. doi: 10.1016/j.amepre.2012.09.043 23253654 10.1016/j.amepre.2012.09.043

[13] International Nordic Walking Association Web site home page [Internet]. 2017 [cited 2017 28/06/2917]. Available from: http://www.inwa-nordicwalking.com/.

[14] Reuter I, Mehnert S, Leone P, Kaps M, Oechsner M, Engelhardt M. Effects of a flexibility and relaxation programme, walking, and nordic walking on Parkinson's disease. J Aging Res. 2011;2011:232473 doi: 10.4061/2011/232473 21603199 10.4061/2011/232473 PMC3095265

[15] Fritschi JO, Brown WJ, van Uffelen JG. On your feet: protocol for a randomized controlled trial to compare the effects of pole walking and regular walking on physical and psychosocial health in older adults. BMC Public Health. 2014;14:375 doi: 10.1186/1471-2458-14-375 24742126 10.1186/1471-2458-14-375 PMC4022438

[16] Willson J, Torry MR, Decker MJ, Kernozek T, Steadman JR. Effects of walking poles on lower extremity gait mechanics. Med Sci Sports Exerc. 2001;33(1):142–7. 11194099 10.1097/00005768-200101000-00021

[17] Homma D, Jigami H, Sato N. Effects of Nordic walking on pelvis motion and muscle activities around the hip joints of adults with hip osteoarthritis. J Phys Ther Sci. 2016;28(4):1213–8. doi: 10.1589/jpts.28.1213 27190455 10.1589/jpts.28.1213 PMC4868215

[18] Porcari JP, Hendrickson TL, Walter PR, Terry L, Walsko G. The physiological responses to walking with and without Power Poles on treadmill exercise. Res Q Exerc Sport. 1997;68(2):161–6. doi: 10.1080/02701367.1997.10607992 9200250 10.1080/02701367.1997.10607992

[19] Church TS, Earnest CP, Morss GM. Field testing of physiological responses associated with Nordic Walking. Res Q Exerc Sport. 2002;73(3):296–300. doi: 10.1080/02701367.2002.10609023 12230336 10.1080/02701367.2002.10609023

[20] Grainer A, Zerbini L, Reggiani C, Marcolin G, Steele J, Pavei G, et al Physiological and Perceptual Responses to Nordic Walking in a Natural Mountain Environment. Int J Environ Res Public Health. 2017;14(10). 10.3390/ijerph14101235 PMC5664736 29039775

[21] Encarnacion-Martinez A, Perez-Soriano P, Llana-Belloch S. Differences in ground reaction forces and shock impacts between nordic walking and walking. Res Q Exerc Sport. 2015;86(1):94–9. doi: 10.1080/02701367.2014.975178 25386664 10.1080/02701367.2014.975178

[22] Girold S, Rousseau J, Le Gal M, Coudeyre E, Le Henaff J. Nordic walking versus walking without poles for rehabilitation with cardiovascular disease: Randomized controlled trial. Ann Phys Rehabil Med. 2017;60(4):223–9. doi: 10.1016/j.rehab.2016.12.004 28347690 10.1016/j.rehab.2016.12.004

[23] Takeshima N, Islam MM, Rogers ME, Rogers NL, Sengoku N, Koizumi D, et al Effects of nordic walking compared to conventional walking and band-based resistance exercise on fitness in older adults. J Sports Sci Med. 2013;12(3):422–30. 24149147 PMC3772584

[24] Pellegrini B, Bortolan L, Schena F. Poling force analysis in diagonal stride at different grades in cross country skiers. Scand J Med Sci Sports. 2011;21(4):589–97. doi: 10.1111/j.1600-0838.2009.01071.x 20459478 10.1111/j.1600-0838.2009.01071.x

[25] Hermens HJ, Freriks B, Disselhorst-Klug C, Rau G. Development of recommendations for SEMG sensors and sensor placement procedures. J Electromyogr Kinesiol. 2000;10(5):361–74. 11018445 10.1016/s1050-6411(00)00027-4

[26] Beretta Piccoli M, Rainoldi A, Heitz C, Wuthrich M, Boccia G, Tomasoni E, et al Innervation zone locations in 43 superficial muscles: toward a standardization of electrode positioning. Muscle Nerve. 2014;49(3):413–21. 24741685 10.1002/mus.23934

[27] Burden A. How should we normalize electromyograms obtained from healthy participants? What we have learned from over 25 years of research. J Electromyogr Kinesiol. 2010;20(6):1023–35. doi: 10.1016/j.jelekin.2010.07.004 20702112 10.1016/j.jelekin.2010.07.004

[28] Zeni JA Jr., Richards JG, Higginson JS. Two simple methods for determining gait events during treadmill and overground walking using kinematic data. Gait Posture. 2008;27(4):710–4. doi: 10.1016/j.gaitpost.2007.07.007 17723303 10.1016/j.gaitpost.2007.07.007 PMC2384115

[29] Smith GK, B & Jakobsen, V, Effectiveness of ski and pole forces in ski skating. 4th International congress on Science and Skiing; 2007; St. Christoph am Arlberg, Austria.

[30] Shiavi R, Frigo C, Pedotti A. Electromyographic signals during gait: criteria for envelope filtering and number of strides. Med Biol Eng Comput. 1998;36(2):171–8. 9684456 10.1007/BF02510739

[31] Richardson JTE. Eta squared and partial eta squared as measures of effect size in educational research. Educational Research Review. 2011;6(2):135–47.

[32] Kocur P, Deskur-Smielecka E, Wilk M, Dylewicz P. Effects of Nordic walking training on exercise capacity and fitness in men participating in early, short-term inpatient cardiac rehabilitation after an acute coronary syndrome—a controlled trial. Clin Rehabil. 2009;23(11):995–1004. doi: 10.1177/0269215509337464 19786418 10.1177/0269215509337464

[33] Ossowski ZM, Skrobot W, Aschenbrenner P, Cesnaitiene VJ, Smaruj M. Effects of short-term Nordic walking training on sarcopenia-related parameters in women with low bone mass: a preliminary study. Clin Interv Aging. 2016;11:1763–71. doi: 10.2147/CIA.S118995 27942207 10.2147/CIA.S118995 PMC5137931

[34] Jurimae T, Meema K, Karelson K, Purge P, Jurimae J. Intensity of Nordic Walking in young females with different peak O2 consumption. Clin Physiol Funct Imaging. 2009;29(5):330–4. doi: 10.1111/j.1475-097X.2009.00872.x 19469785 10.1111/j.1475-097X.2009.00872.x

[35] Grimmer K. An investigation of poor cervical resting posture. Aust J Physiother. 1997;43(1):7–16. 11676668 10.1016/s0004-9514(14)60398-6

[36] Andersen CH, Andersen LL, Zebis MK, Sjogaard G. Effect of scapular function training on chronic pain in the neck/shoulder region: a randomized controlled trial. J Occup Rehabil. 2017;24(2):316–24. 10.1007/s10926-013-9441-1 PMC4000422 23832167

[37] Saeterbakken AH, Nordengen S, Andersen V, Fimland MS. Nordic walking and specific strength training for neck- and shoulder pain in office workers: a pilot-study. Eur J Phys Rehabil Med. 2017. 10.23736/S1973-9087.17.04623-8 28569455

[38] Kocur P, Wiernicka M, Wilski M, Kaminska E, Furmaniuk L, Maslowska MF, et al Does Nordic walking improves the postural control and gait parameters of women between the age 65 and 74: a randomized trial. J Phys Ther Sci. 2011;27(12):3733–7. 10.1589/jpts.27.3733 PMC4713780 26834341

[39] Kuhtz-Buschbeck JP, Jing B. Activity of upper limb muscles during human walking. J Electromyogr Kinesiol. 2012;22(2):199–206. doi: 10.1016/j.jelekin.2011.08.014 21945656 10.1016/j.jelekin.2011.08.014

[40] Padulo J, Maffulli N, Ardigo LP. Signal or noise, a statistical perspective. Proc Natl Acad Sci U S A. 2013;111(13):E1160. 10.1073/pnas.1400112111 PMC3977304 24578511

